# Endotrophin – Linking Obesity with Aggressive Tumor Growth

**DOI:** 10.18632/oncotarget.796

**Published:** 2012-12-20

**Authors:** Jiyoung Park, Philipp E. Scherer

**Affiliations:** Touchstone Diabetes Center, Department of Internal Medicine, The University of Texas Southwestern Medical Center, Dallas, Texas, USA; Touchstone Diabetes Center, Departments of Internal Medicine, Cell Biology and Simmons Cancer center, The University of Texas Southwestern Medical Center, Dallas, Texas, USA

**Figure 1:**
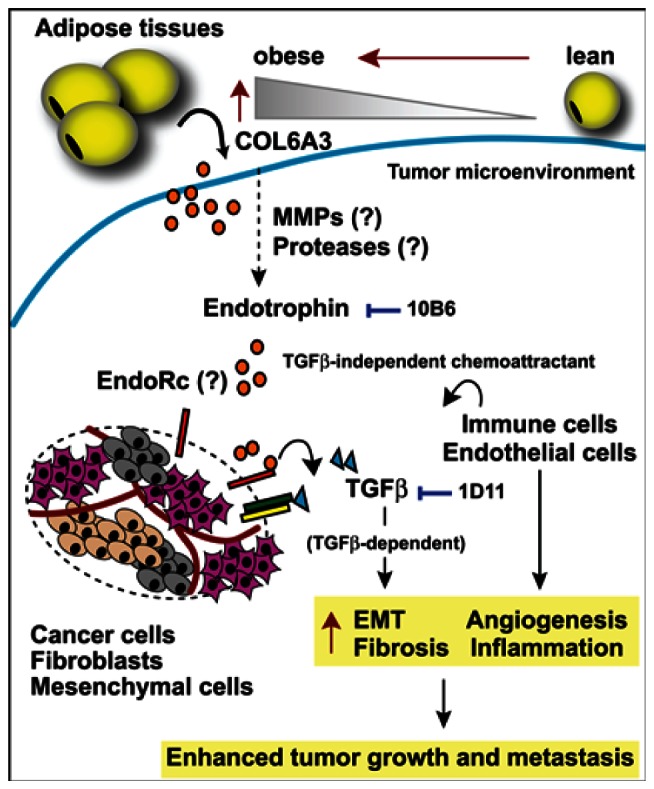
Endotrophin-mediated changes in the tumor stroma Adipocyte-derived COL6A3 levels are increased during obesity. Endotrophin is cleaved from the COL6A3 parent chain within the tumor microenvironment. Endotrophin potentiates TGFβ-dependent epithelial-mesenchymal transition (EMT) and fibrosis and displays chemoattractive activity, recruiting endothelial cells and macrophages, leading to enhanced angiogenesis and chronic inflammation. All of these activities induced by endotrophin synergistically lead to enhanced tumor growth and metastasis. Either the endotrophin neutralizing monoclonal antibody (10B6) or the TGFβ antagonizing monoclonal antibody (1D11) differentially attenuate a subset of endotrophin effects. EndoRc: endotrophin receptor.

Collagen VI (COL6, encoded by the COL6A1, COL6A2, and COL6A3 genes) is an extracellular matrix protein that forms a microfilamentous network in various connective tissues, including skeletal muscle, cartilage, skin and adipose tissue. Among the various tissues, adipose tissue is by far the most abundant source of COL6 microfilaments [1]. Clinically, mutations in COL6 develop mild muscle myopathies (such as Bethlem myopathy and Ullrich congenital muscular dystrophy), with symptoms of muscle weakness and apoptosis combined with joint hyperlaxity and contractures [2]. A genetically engineered mouse model, deficient in COL6 microfilament formation and secretion, has been widely used to investigate the roles of COL6 under physiological and pathological conditions. COL6 deficiency in mice leads to the development of muscle dystrophies resembling Bethlem myopathy in man [3]. In the area of tumor biology, COL6 has been identified as a tumor-promoting factor abundantly produced and released from adipocytes [4]. Subsequent analysis of the COL6 functional null mice bred into the murine MMTV-PyMT mammary tumor model (mouse mammary tumor virus-polyoma middle T antigen) showed a significant attenuation of early onset mammary tumor progression [5]. Specifically, the carboxyl-terminal domain of the COL6A3 chain is massively upregulated in the malignant tumors of human patients compared to the remaining part of COL6A3 chain [5]. We recently followed up on this phenomenon and demonstrated that the cleavage product from the carboxyl-terminus of the COL6A3 chain (that we refer to as *endotrophin*) accounts for the tumor-promoting effects associated with COL6 [6]. Ectopic expression of the isolated endotrophin fragment within the tumor microenvironment of MMTV-PyMT mice drives an increase of both primary tumor growth and pulmonary metastasis through an enhancement of the expansion of the tumor stroma [6]. Additional prominent effects associated with endotrophin overexpression in the tumor stroma include an increase in fibrosis, angiogenesis and inflammation through increased fibrogenesis, a stimulation of epithelial-mesenchymal transition (EMT) and chemokine activities; these are well-established stromal phenomena that support aggressive traits of tumors (Figure 1). Indeed, neutralizing monoclonal antibodies against endotrophin suppress tumor growth and reduce metastatic growth in MMTV-PyMT mice [6]. EMT of tumors conveys metastatic traits and multiple drug resistances to cancer cells. Since endotrophin is a potent stimulator of EMT, it suggests that the neutralization of endotrophin may lend itself to enhance chemo-sensitivity in combination with conventional therapeutic regimens, though this remains to be directly shown.

Adipose tissue is a crucial organ for the maintenance of whole body energy homeostasis, and also a major source of COL6. We have therefore explored the roles of COL6 in metabolic homeostasis even without a tumor burden. Metabolic characterization of the COL6A1 functional null mice bred with a genetically obese animal model, the *ob/ob* mouse, reveals that COL6 deficiency improves systemic metabolic profiles, including enhanced insulin sensitivity and glucose metabolism. This is likely due to a number of changes, but the reduced fibrotic stress commonly seen in hypertrophic adipose tissues in obese status is likely to be a contributing factor [7]. COL6 is upregulated in obese and dysfunctional adipose tissue, and anti-diabetic treatment regimens lead to a suppression of COL6 expression. Tumor lesions in the microenvironment lead to a further local enrichment of endotrophin, either through stimulation of syntheses and/or cleavage of endotrophin from the mature protein, or through an induction of production within the tumor lesions themselves. As such, endotrophin is likely to constitute one of the risk factors that mediate the more aggressive lesion growth and worse prognosis seen in patients with higher body mass indices (BMIs). More importantly, it is likely that endotrophin plays a pro-fibrotic and pro-inflammatory role in a number of additional tissues, even in the absence of a tumor challenge. This may be relevant for adipose tissues, liver and kidney, all tissues that are prone to fibrosis and chronic inflammation under pathological conditions. Therefore, inhibition of endotrophin activity under such pathological conditions is likely to be associated with clinical improvements.

Two important issues remain to be resolved. These include the question as to which matrix metalloproteinase(s) (MMPs) specifically cleave endotrophin from its parent COL6A3 chain. Furthermore, a second important question is which cell surface proteins act as receptors to mediate endotrophin action, leading to downstream signaling events triggering the enhanced TGFβ signaling and the chemokine action. Answers to these questions will have a significant impact on our understanding of the significance of endotrophin under normal and pathological conditions.
